# Quantitative evaluations of variations using the population mean as a baseline for bioinformatics interpretation

**DOI:** 10.7717/peerj.14955

**Published:** 2023-02-24

**Authors:** Liu Hui

**Affiliations:** College of Medical Laboratory, Dalian Medical University, Dalian, China

**Keywords:** Evaluation, Bioinformation, Variation, Analytical model

## Abstract

**Objective:**

The purpose of this study were to establish a model of quantitative evaluation that uses the population mean as a baseline of variations and describe variations derived from different types and systems using new concepts.

**Methods:**

The observed datasets, including measurement data and relative data, were transformed to 0–1.0 using the population mean. Datasets derived from different types (same category of dataset, different categories of datasets, and datasets with the same baseline) were transformed using different methods. The ‘middle compared index’ (MCI) was used to describe the change in magnitude as follows: [a/(a+b)+(1−b)/(2−a−b)−1]^1.7^, where ‘a’ represents the number after the magnitude change and ‘b’ represents the number before the magnitude change. Actual data were used to observe the MCI’s ability to evaluate variations quantitatively.

**Results:**

When the value before the magnitude change was equal to that after the magnitude change, the MCI was equal to 0; when the value before the magnitude change was equal to 0 and that after the magnitude change was equal to 1, the MCI was equal to 1. This implies the MCI is valid. When the value before the magnitude change was 0 and that after the magnitude change was 0.5, or when the value before the magnitude change was 0.5 and that after the magnitude change was 1.0, each MCI was approximately equal to 0.5. The values derived from the absolute, ratio, and MCI methods were different, indicating that the MCI is an independent index.

**Conclusion:**

The MCI perfectly performs as an evaluation model using the population mean as the baseline, and it may be more a reasonable index than the ratio or absolute methods. The MCI increases our understanding of quantitative variations in evaluation measures of association using new concepts.

## Introduction

Quantitative variations in a particular event are normally distributed in terms of changes in ratio (Lemans et al., 2019; Zhu & Wu, 2019; Zhou, Sun & Liu, 2019) and absolute values (Scribani et al., 2019; Hydes et al., 2019), although these factors are not comparable (Hongwei et al., 2020; Xiaojun & Hui, 2019). When the cardinal number of a ratio is relatively small, an increasing ratio may be very high although the absolute increase may be not high. In contrast, when the cardinal number of a ratio is relatively large, an increasing ratio may not be high although the absolute increase may be highly significant. If the probability of the occurrence of a gene is 0.2 in the disease group and 0.05 in the control group, its ratio is 4.0 (0.2/0.05), while its absolute value is 0.15 (0.2–0.05). If the probability of the occurrence of another gene is 0.3 in the disease group and 0.1 in the control group, its ratio is 3.0 (0.3/0.1), while its absolute value is 0.2 (0.3–0.1). Therefore, if a ratio is used as a measure or index of change, the variation of the first gene would be greater. However, if the absolute change in value is used as an index of change, the variation of the latter gene is greater. The problem is that the ratio method only reflects relative change using the cardinal number as the baseline value, whereas the difference method only reflects the absolute change using zero as the cardinal number as the baseline. The difference method does not take the cardinal number into account and is not useful for comparing different categories of events. When the cardinal number is zero, the ratio method cannot be used to describe relative change.

For continuous variable, the difference before and after the change can usually be described using the standard deviation of that group. However, the parameters describing the change in continuous variables are not comparable with the parameters of binary variables, which in turn leads to incomparable changes in different types of indicators, affecting the judgement and comprehension of the observation. This presents a serious problem for comparing variations in the quantity of events described by different cardinal numbers. Therefore, new methods and indices are required for these purposes. At present, few research reports have addressed this problem. Here, we propose models that use 50% (the population mean) as a reference point and a new index (the middle compared index, MCI) to quantitatively evaluate variations, which has the potential to solve the above problem.

## Methods

The first principle of this study is that one should use the population mean as a reference point to assess variation quantitatively. The observed dataset, including measurement data and relative data, should be transformed into a 0–1.0 scale based on the population mean so that the population mean is 0.5.

### Transformation of the same category of datasets

The probability of the occurrence of a gene in the two groups mentioned in the Introduction falls into the same category of dataset. When this is true, the observed value (X) should be transformed according to the following formula:

(1) $$X^\prime = \bigg(\arctan \displaystyle{X \over {\bar X}}\bigg)/90$$

where X′ represents the transformed value; X′ ranges from 0 to 1. When the observed value is equal to the average value, the transformed value is 0.5. For Eq. (1), only the population mean is needed to complete the transformation; therefore, arctan method involves few parameters and is easy to use.

### Transformation of different categories of datasets

Comparisons of different indices, such as changes in the magnitude of triglyceride and cholesterol, are different categories of comparisons. The transformation of different categories of datasets should calculate the mean and standard deviation (SD) as follows:

(2) $$X^\prime = e^{(X - \bar X)/SD}{\left/ {\big[1 + {e^{(X - \bar X)/SD}}\big]}\right.}$$

where X′ represents the transformed value; X′ ranges from 0 to 1. When the observed value is equal to the average value, the transformed value is 0.5.

### Transformation of datasets with the same baseline according to the evaluation model

Biological sample measurement usually use a colourimetric method to quantify the substances measured in the sample, as enzyme-linked immunosorbent assay (ELISA). A blank control is usually set up to measure the background value, which needs to be subtracted when the measured value is read. The subtraction of background values can be done either by the ratio or the difference method, in this article MCI is recommended. Selection of the optimal conditions for ELISA is used as an example for the transformation of datasets with the same baseline.

We added 50 μl of the colour developing reagent (enzymatic substrate) to the positive well and negative well in the last step of ELISA. Then, the ELISA plate was shaken for 20 s, and placed in a dark room until it reached a ‘satisfactory colour’ over time. Next, the optical density (OD) was measured at 450 nm. Theoretically, there should be no enzymes in the negative well; however, if there was still a small amount of enzymes in the negative well, a slight coloration would be present in the negative well. Therefore, the ‘satisfactory colour’ would be the ratio or the absolute OD between the positive well and the negative well, when the baselines of both the positive well and the negative well were zero. The OD values were determined at 30 and 60 min after the addition of the colour developing reagent. The observed OD values were 0.296 and 0.082, respectively, for the positive well and negative well at 30 min, and 0.565 and 0.175, respectively, for the positive well and negative well at 60 min. The data were initially transformed at 2 times the maximum number, as follows:

(3) $$X^\prime = \displaystyle{X \over {2{X_{\max }}}}$$

The denominator was two times the maximum number (2 × 0.565); thus, the above data were changed to percentages, which were 0.500 (0.565/1.130), 0.155 (0.175/1.130), 0.262 (0.296/1.130), and 0.073 (0.082/1.130), respectively.

### The middle compared index (MCI)

**Figure 1:**
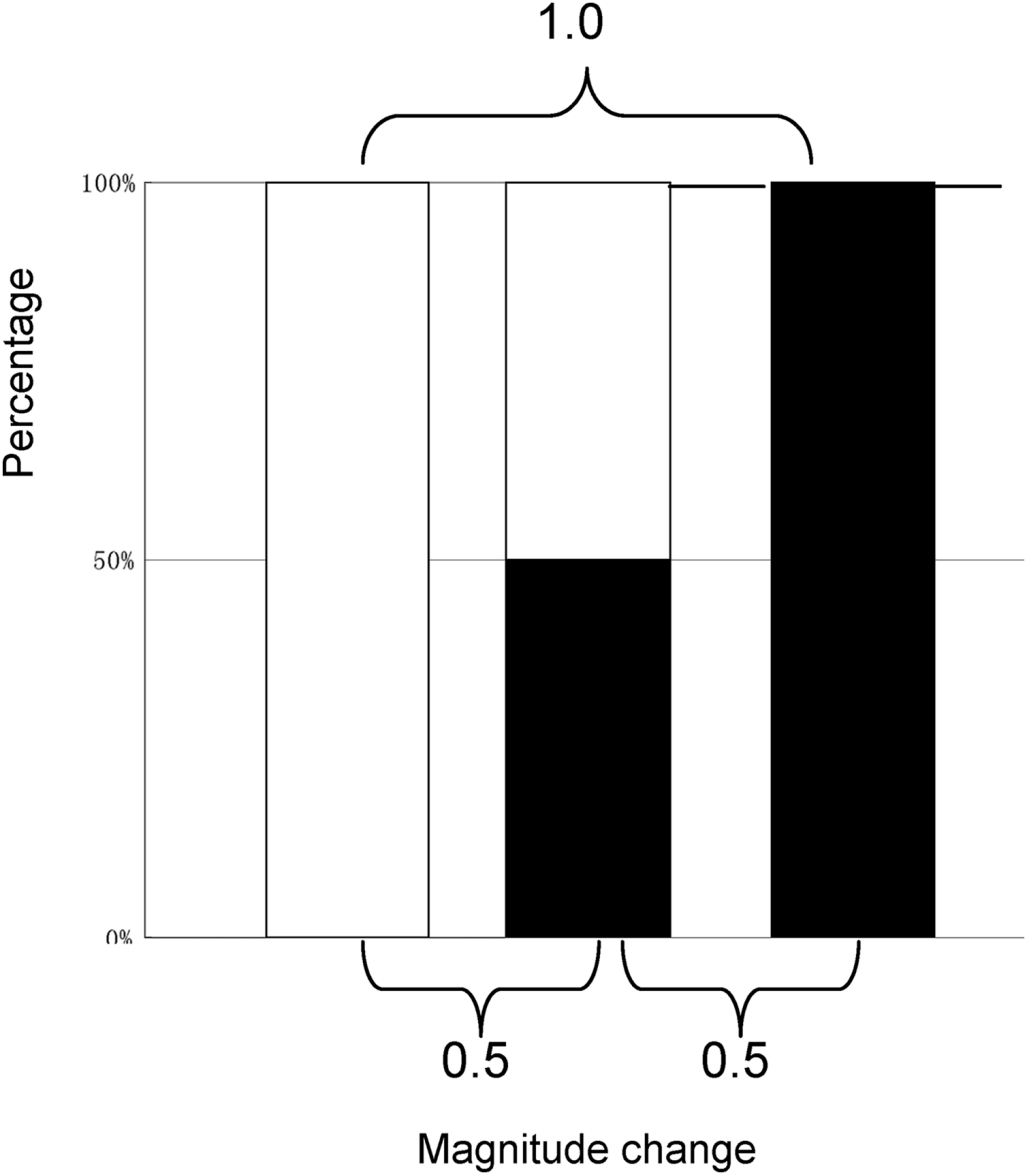
The model for the quantitative evaluation of variation.

The MCI evaluation model was based on the following assumptions: (1) the range of magnitude change would be 0–1.0; (2) 0.5 would be considered the baseline to assess variation; (3) the magnitude change would be equaled between (0.5−X, X < 0.5) – 0.5 and 0.5 – (0.5+X); and (4) the outcome of magnitude change from 0 to 0.5 or that from 0.5 to 1.0 would be 0.5, as close as possible to the absolute change needed for quantitative evaluation (see Fig. 1).

Based on these assumptions, the MCI was calculated as follows:

(4) $$MCI = {\bigg(\displaystyle{a \over {a + b}} + \displaystyle{{1 - b} \over {2 - a - b}} - 1\bigg)^{1.7}}$$

where ‘a’ represents the number after the magnitude change and ‘b’ represents the number before the magnitude change. When a = b, a/(a+b) is 0.5 and (1−b)/(2−a−b) is also 0.5, 0.5 can be considered to be the baseline of the MCI. When a = 0.5, b = 0 or a = 1, b = 0.5; the MCI = 0.667^1.7^ = 0.502, which approximately corresponds to the evaluation model (MCI = 0.500); thus, it should be a power of 1.7. It is suggested that the MCI can describe relative change even if the cardinal number is zero, also suggesting that the MCI corresponds to the above evaluation model. Given the possible range of an MCI (0–1.0), an observed MCI of 0.25–0.5 implies medium variation.

Because datasets with the same baselines can be transformed at two times the maximum number, the range of transformed data should be 0–0.5, and the range of the MCI derived from datasets with a same baseline MCIs should also be 0–0.5. Therefore, MCIs should be multiplied by 2:

(5) $$\rm {MCIs = MCI \times 2}$$

Thus, the range of MCIs would be 0–1.0, approximately, and MCIs would be comparable with other MCIs.

### Actual data using the MCI to quantitatively evaluate variations

Schizophrenia (SCH) is a typical polygenic disease (Liu et al., 2007; Guang et al., 2014). Hence, genetic factors appear to play an important role in the onset of SCH. We used raw data obtained from our previous work to give an example of transforming data from the same category, according to the conditions of the MCI.

Biological variation of laboratory indices is the inherent physiologically promulgated variations in an individual (Johnson, Shahangian & Astles, 2021; Carobene et al., 2021; Hui et al., 2009). Our previous work on biological variations of laboratory indices provided an example of transforming different categories of datasets, according to an evaluation model for observing which index has the greatest biological variation, using the MCI.

The selection of the optimal conditions for ELISA, mentioned above, was used as an example for transforming datasets with the same baseline. Based on MCI, it was possible to determine whether 30 or 60 min is better for colour development.

## Results

### MCI evaluation

**Figure 2:**
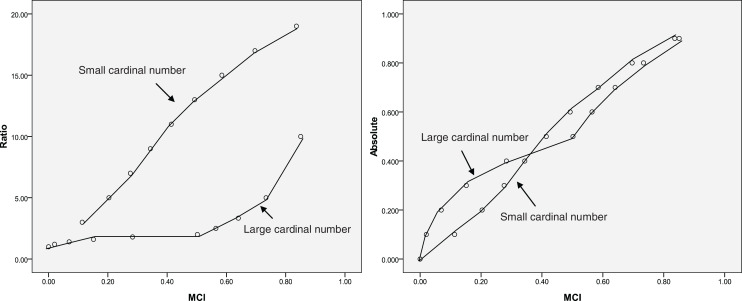
Relationship between the middle compared index (MCI), the ratio and the absolute index (same MCI correspond to different ratio or absolute values).

A special dataset is presented in Table 1 that reflects how well MCI fits the MCI evaluation model (Fig. 1) and the relationships among absolute change, ratio change, and MCI change could help understand the change described by the MCI. As shown in Table 1, the influence of zero on relative change is eliminated using the MCI. When a = 0.5, b = 0 or a = 1, b = 0.5; the MCI = 0.50, suggesting that the MCI corresponds to the evaluation model. The results also show that the MCI increases with increasing ratio and absolute change; however, same MCI correspond to different ratio or absolute values, as shown in Fig. 2. This indicates that the MCI is independent index.

**Table 1:** A special dataset for observing the MCI corresponding to evaluation models.

Cardinal number	Number		Variation		MCI (middle compared index)
	After magnitude	Before magnitude	Ratio	Absolute	
	**0.50**	**0.00**	–	**0.50**	**0.50**
	**1.00**	**0.00**	–	**1.00**	**1.00**
	0.15	0.05	3.00	0.10	0.11
	0.25	0.05	5.00	0.20	0.20
Small	0.35	0.05	7.00	0.30	0.28
	0.45	0.05	9.00	0.40	0.34
	0.55	0.05	11.00	0.50	0.41
	0.65	0.05	13.00	0.60	0.49
	0.75	0.05	15.00	0.70	0.58
	0.85	0.05	17.00	0.80	0.70
	0.95	0.05	19.00	0.90	0.84
	0.50	0.50	1.00	0.00	0.00
	0.60	0.50	1.20	0.10	0.02
	0.70	0.50	1.40	0.20	0.07
Large	0.80	0.50	1.60	0.30	0.15
	0.90	0.50	1.80	0.40	0.28
	**1.00**	**0.50**	**2.00**	**0.50**	**0.50**
	1.00	0.40	2.50	0.60	0.56
	1.00	0.30	3.33	0.70	0.64
	1.00	0.20	5.00	0.80	0.73
	1.00	0.10	10.00	0.90	0.85

**Note:**

The numerical value derived from evaluation model (Fig. 1) is shown in bold..

### Comparison of same category of dataset using the MCI

We use the evaluation of which gene has the most effect on SCH as an example. Our previous study found the frequency of HLADRB1*07-02 was 0.040 in the group with SCH and 0.013 in the control group (*p* = 0.002) (Laiyu et al., 2017). We also found the frequency of HLADQB1*03 was 0.533 in the schizophrenic group and 0.420 in the control group (*p* = 0.035) (Laiyu et al., 2017), as shown in Table 2. The results revealed that the MCI was 0.109 for HLADRB1*07-02 and 0.011 for HLADQB1*03, which indicates that the HLADRB1*07-02 gene had a greater variation in SCH.

**Table 2:** Evaluation of what gene has more effect on schizophrenia (SCH).

Genes	Original data				Transformed data^1^		
	SCH	Control	Ratio	Absolute	SCH	Control	MCI^2^
HLADRB1*03	0.533	0.420	1.269	0.113	0.718	0.657	0.011
HLADQB1*07-02	0.040	0.013	3.077	0.027	0.101	0.033	0.109

**Notes:**

1 Original data transformed by Eq. (1).

2 MCI, middle compared index, calculated by Eq. (4).

### Comparison of different categories of datasets using the MCI

We use the evaluation of which laboratory indices have greater biological variation as an example. The results of our previous work on biological variation are shown in Table 3 (Guang, Yuzhong & Hui, 2022), which show the observed mean and standard deviation (SD) obtained from the reference range (normal distribution). The results revealed that the MCI was 0.006 for albumin and 0.005 for urea, implying that albumin exhibits greater biological variation than urea does.

**Table 3:** Evaluation of what laboratory indices have greater biological variation.

Indices	Reference range	Mean ± SD	Original data			Transformed data^1^		
			Day 1	Day 2	Ratio	Day 1	Day 2	MCI^2^
Albumin	40~50	45 ± 2.5	47.0	47.5	1.01	0.69	0.73	0.006
Urea	2.8~7.1	5.0 ± 1.1	3.9	4.1	1.02	0.86	0.88	0.005

**Notes:**

1 Original data transformed by Eq. (2).

2 MCI, middle compared index, calculated by Eq. (4).

### Comparison of datasets with the same baseline

We use the evaluation of what is the optimal condition for ELISA, as mentioned above, as an example. The OD value was determined at 30 and 60 min after the addition of the colour developing reagent (see Table 4). A larger score indicates a ‘satisfactory colour’. The results showed that it was possible to determine that 60 min was better than 30 min, based on the MCI.

**Table 4:** Evaluation of what is the optimal condition for ELISA.

Time	OD value		Ratio	Absolute	Transformed data^1^		
	Negative	Positive			Negative	Positive	MCI^2^
30 min	0.082	0.296	3.610	0.241	0.073	0.262	0.334
60 min	0.175	0.565	3.229	0.390	0.155	0.500	0.454

**Notes:**

1 Original data transformed by Eq. (3).

2 MCI, middle compared index, calculated by Eqs. (4) and (5).

## Discussion

The purpose of the quantitative description of variations is to make comparisons. Quantitative variations are normally described in terms of changes in ratio values and absolute values. The ratio method uses an index derived from a own cardinal number as the baseline, whereas the difference method uses zero derived from a cardinal number as the baseline. Therefore, the values of the ratio and absolute method indices are not comparable. The present study proposed a model that uses the population mean as a reference point to create a new index (the MCI) for quantitatively evaluating variations in measurement data and relative data.

A transformation is performed using the population mean for the arrays to be compared. Different datasets require the use of different methods. The range of the transformed data is 0–1.0 to establish a quantitative evaluation model in which 0.5 should be considered the baseline to assess variation. On the other hand, one key assumption for this mean-based transformation is the original data should follow a normal distribution. When dealing with a dataset that fails the Shapiro-Wilk test, the normal distribution should be adjusted before transformation.

Theoretically, as the index increases from 0% to 100%, the increase in magnitude is maximised; the MCI also provides the maximum score at this time (Table 1). When the index does not change or the index is the same before and after the comparison (the absolute change is zero), the MCI yields a score of zero. This suggests that the MCI is valid. The results also show that the same MCI correspond to different ratio or absolute values (Fig. 2), indicating that the MCI is independent index.

When the value before magnitude change is zero and that after the magnitude change is 0.5 or 1.0, the MCI produces different values, implying that the influence of the zero on the relative change has been eliminated using the MCI. When the value before the magnitude change is zero and that after the magnitude change is 0.5, or when that of value is 0.5, and that after the magnitude change is 1.0, each MCI approximately produces 0.5, implying that the MCI is reasonable and also suggesting that the MCI can perfectly conform to an evaluation model.

In fact, the MCI is also a relative index (taking the population mean as the denominator). The results showed that the MCI increases with increasing absolute change and there is a linear relationship between the MCI and absolute change, suggesting that the MCI could be a hybrid index that reflects changes in ratio and absolute indices to describe variations. The results also showed that values derived from absolute, ratio, and MCI methods were different, suggesting that the MCI is an independent index. An ‘MCI = 0.5’ can be understood as a magnitude between 0 and 0.5, or between 0.5 and 1.0, as shown in Fig. 1, which implies that the MCI can be considered a common-sense judgment system for intuitively understanding variations. The author proposed that an MCI score <0.25 indicates low variation, an MCI score of 0.25–0.5 indicates medium variation, and an MCI score above 0.5 indicates a significantly high level of variation.

Analyses using actual data revealed that values derived from absolute, ratio, and MCI methods were different. The results revealed that the MCI of SCH was 0.109 for HLADRB1*07-02, indicating that the HLADRB1*07-02 gene had a greater variation in SCH. However, Absolute of SCH was 0.113 for HLADRB1*03, indicating that the HLADRB1*03 gene had a greater variation in SCH.

We use the evaluation of which laboratory indices have greater biological variation. The results revealed that the MCI was 0.006 for albumin and 0.005 for urea, implying that albumin exhibits greater biological variation than urea does. However, ratio of urea was 1.02, suggesting that urea exhibits greater biological variation.

We also use the evaluation of what is the optimal condition for ELISA. The results showed that it was possible to determine that 60 min was better than 30 min, based on the MCI as shown in Table 4 and suggesting a medium variation. However, it was possible to determine that 30 min was better than 60 min, based on the ratio method.

The MCI, which uses the population mean as a baseline, may be more reasonable than the ratio or absolute methods, which use a own cardinal number as their baselines. Thus, the MCI enhances our understanding of the quantitative variations in evaluation measures of association, using new concepts.

It should be noted that the MCI differs from effect size. Effect size is a ratio of the outcome explained by the observed factor (Xia et al., 2019). The MCI also differs from the risk from a factor. Risk is the probability of the occurrence of an event from a causal factor (Lemans et al., 2019; Zhu & Wu, 2019; Scribani et al., 2019; Hydes et al., 2019). Nor is the MCI the same as a diagnostic effect, as a diagnosis considers consistent rates, including both observed consistency and predicted consistency. A comprehensive index of biomarkers based on our previous work is recommended for evaluating diagnostic effects (Hui, 2021). Although a higher MCI may imply a higher effect or risk from an observed factor, the MCI is only used for quantitatively describing the variations for comparisons; it does not evaluate the causes of variation.

In conclusion, the primary significance of this study is that we established a model of quantitative evaluation that uses the population mean as a baseline of variations with different cardinal numbers derived from different types and systems. The MCI can perfectly perform as an evaluation model and may be more reasonable than ratio and absolute models. The MCI further increases our understanding of the quantitative variations in evaluation measures of association, using new concepts.

## Additional Information and Declarations

### Data Availability

The following information was supplied regarding data availability: The previously reported data used to support this study is available at: Guang Y, Yuzhong L, Hui L. Evaluation of Biomarkers from Routine Laboratory Indicators for Establishing Molecular Age Score with Considering Survival Probability. 2022. Clinical Laboratory 68(5) DOI 10.7754/Clin.Lab.2021.210712. Laiyu S, Lei Z, Soufu X, Junting X, Jie Z, Hui L. A Cross-Validation Design Using Age and HLA Alleles for the Assessment of Clinical Schizophrenia Risk. 2017 Clinical Laboratory 63(2):255–260. DOI 10.7754/Clin.Lab.2016.160725.
